# Defining hypotension in the emergency department and in the pre-hospital setting: A hospital-based cohort study

**DOI:** 10.1186/1757-7241-23-S1-A11

**Published:** 2015-07-16

**Authors:** Anders KB Kristensen, Jon G Holler, Søren Mikkelsen, Jesper Hallas, Annmarie Lassen

**Affiliations:** 1 Department of Emergency Medicine, OUH Odense University Hospital, Odense, Denmark; 2 Department of Anaesthesiology and Intensive Care Medicine, OUH Odense University Hospital, Odense, Denmark; 3 Clinical Pharmacology, Institute of Public Health, University of Southern Denmark, Odense, Denmark

## Background

Systolic blood pressure is a key parameter when identifying patients in shock. However, the systolic blood pressure level below which a given patient should be considered hypotensive is subject to debate. Furthermore, recent studies have advocated higher systolic blood pressure thresholds than the traditionally recognized 90 mmHg. The aim of this study was to identify the best performing systolic blood pressure thresholds with regards to predicting 7-day mortality and to evaluate the applicability of these in the emergency department and in the pre-hospital setting.

## Methods

A hospital-based cohort study from Odense University Hospital of all adult patients in the emergency department between 1995 and 2011, all patients transported to the emergency department in non-physician staffed ambulances in the period 2012-2013, and all patients serviced by the physician staffed ambulances in Odense between 2007 and 2013. Exposure was the first recorded systolic blood pressure and the main outcome was 7-day mortality. Best performing thresholds were identified with methods based on receiver operating characteristics and multivariate regression. Performance of systolic blood pressure thresholds was evaluated with standard summary statistics for diagnostic tests.

## Results

7-day mortality rates varied from 1.8% (95% CI [1.7, 1.9]) of 112,727 patients in the emergency department to 2.2% (95% CI [2.0, 2.5]) of 15,862 patients in the non-physician staffed ambulance and 5.7% (95% CI [5.3, 6.2]) of 12,270 patients in the physician staffed ambulance cohort. Best performing thresholds ranged from 95 to 119 mmHg in the emergency department, 103-120 mmHg in the non-physician staffed ambulance, and 101-115 mmHg in the physician staffed ambulance.

## Conclusions

A systolic blood pressure threshold of 100-110 mmHg might be a clinically relevant trigger point in the emergency department and the pre-hospital setting.

